# Communicating with conscious and mechanically ventilated critically ill patients: a systematic review

**DOI:** 10.1186/s13054-016-1483-2

**Published:** 2016-10-19

**Authors:** S. ten Hoorn, P. W. Elbers, A. R. Girbes, P. R. Tuinman

**Affiliations:** 1 Department of Intensive Care Medicine and Research VUmc Intensive Care (REVIVE), VU University Medical Center Amsterdam, Room ZH—7D-166, De Boelelaan 1117, PO Box 7057, Amsterdam, 1007 MB The Netherlands; 2 Institute for Cardiovascular Research VU (ICaR-VU), VU University Medical Center Amsterdam, Amsterdam, The Netherlands

**Keywords:** Intensive care, Communication intervention, Communication tools, Communication methods, Mechanical ventilation

## Abstract

**Background:**

Ventilator-dependent patients in the ICU often experience difficulties with one of the most basic human functions, namely communication, due to intubation. Although various assistive communication tools exist, these are infrequently used in ICU patients. We summarized the current evidence on communication methods with mechanically ventilated patients in the ICU. Secondly, we developed an algorithm for communication with these patients based on current evidence.

**Methods:**

We performed a systematic review. PubMed, Embase, Cochrane, Cinahl, PsychInfo, and Web of Science databases were systematically searched to November 2015. Studies that reported a communication intervention with conscious nonverbal mechanically ventilated patients in the ICU aged 18 years or older were included. The methodological quality was assessed using the Quality Assessment Tool.

**Results:**

The search yielded 9883 publications, of which 31 articles, representing 29 different studies, fulfilled the inclusion criteria. The overall methodological quality varied from poor to moderate. We identified four communication intervention types: (1) communication boards were studied in three studies—they improved communication and increased patient satisfaction, but they can be time-consuming and limit the ability to produce novel utterances; (2) two types of specialized talking tracheostomy tubes were assessed in eight studies—audible voicing was achieved in the majority of patients (range 74–100 %), but more studies are needed to facilitate safe and effective use; (3) an electrolarynx improved communication in seven studies—its effectiveness was mainly demonstrated with tracheostomized patients; and (4) “high-tech” augmentative and alternative communication (AAC) devices in nine studies with diverse computerized AAC devices proved to be beneficial communication methods—two studies investigated multiple AAC interventions, and different control devices (e.g., touch-sensitive or eye/blink detection) can be used to ensure that physical limitations do not prevent use of the devices. We developed an algorithm for the assessment and selection of a communication intervention with nonverbal and conscious mechanically intubated patients in the ICU.

**Conclusions:**

Although evidence is limited, results suggest that most communication methods may be effective in improving patient–healthcare professional communication with mechanically ventilated patients. A combination of methods is advised. We developed an algorithm to standardize the approach for selection of communication techniques.

**Electronic supplementary material:**

The online version of this article (doi:10.1186/s13054-016-1483-2) contains supplementary material, which is available to authorized users.

## Background

Communication with hospitalized patients is essential to improve the quality and safety of health care [1]. Patients in the ICU are often deprived of speech and their ability to communicate, because of intubation. There is a significant relationship between the loss of speech and severe emotional reactions among ICU patients, such as a high level of frustration, stress, anxiety, and depression [2–10]. The most commonly used communication methods with critically ill patients, like lip reading, gestures, and head nods [7, 8, 11–13], are time-consuming, inadequate to meet all communication needs, and frustrating for both patients and nurses [12–19]. Current practice in the ICU is to use less sedation in mechanically ventilated patients, which increases the number of patients potentially able to communicate while mechanically ventilated and awake [2, 20]. Even though there are numerous alternative methods of communication available and about 50 % of the ICU patients could potentially be served by simple assistive communication tools [21], caregivers currently make little to no use of the devices for patients in the ICU [7, 8, 22].

Improving communication could be achieved by using a communication algorithm to standardize the approach of selecting various augmentative and alternative communication (AAC) methods. AAC refers to all forms of communication, other than oral speech, that are used to express messages [23]. Although considering the aformentioned importance of adequate communication for the quality of patient care and well-being, to our knowledge and based on an extensive search of websites for societies of intensive care medicine (Dutch Society of Intensive Care Medicine; European Society of Intensive Care Medicine; World Federation of Societies of Intensive and Critical Care Medicine; Society of Critical Care Medicine; Intensive Care Society) and critical care nursing (American Association of Critical-Care Nurses; European Federation of Critical Care Nursing Associations; Canadian Association of Critical Care Nurses; Australian College of Critical Care Nurses), no protocols or guidelines about communication with intubated patients in the ICU currently exist.

The aim of our systematic review was to summarize the current published evidence on communication methods used with adult nonverbal mechanically ventilated patients in the ICU. Our secondary objective was to develop an algorithm for a structured approach of assistive communication devices with mechanically ventilated patients.

## Methods

### Search strategy, data sources, and study selection

A systematic electronic search was conducted in PubMed, Embase, Cochrane, Cinahl, PsychInfo, and Web of Science databases (to November 2015). The screening of titles and abstracts was done by two independent reviewers. Additional file 1 shows our detailed search strategy. We hand-searched the reference lists of retrieved papers for additional studies. Furthermore, we screened the Internet for the registered clinical studies (clinicaltrials.gov). We included all randomized clinical trials, quasi-experimental studies, and observational studies published in English. The population under consideration included all adult patients in the ICU who were conscious with an oral tube or tracheostomy with inflated cuff. Because we aimed for patients who were completely ventilator dependent, we excluded patients who could tolerate cuff deflation. The interventions that are used when the cuff is deflated were therefore excluded (e.g., one-way speaking valves). The main focus of the studies had to be the use of communication techniques between healthcare professionals and patients, not the information content. We adapted our search strategy during the search process, in close collaboration with a medicine literature search specialist of the Free University Medical Library, and allowed any control group and outcome measure due to the minimal research on the subject. Published conference abstracts with no full article were excluded. Identified titles and abstracts were screened against the inclusion criteria to find potentially relevant papers, and subsequently the full texts were reviewed against the eligibility criteria already described. Any disagreements about study selection were resolved during consensus meetings.

### Data extraction and quality assessment

A data collection form was used to abstract the data from included articles and assess the study quality. Both the abstracted data and the study quality were checked by a second reviewer. To assess the methodological quality and risk of bias of individual studies, we used the Quality Assessment Tool (QATSDD). This tool can be applied to a diverse range of study designs, including qualitative and quantitative methods, and has clear defined scales. The QATSDD consists of 16 criteria, which all apply to mixed-methods studies. There are also 14 criteria that apply to qualitative studies and 14 criteria that apply to quantitative studies. Test–retest and inter-rater reliabilities have been assessed and ranged from good to substantial (kranging from 0.698 to 0.901) [24].

### Data synthesis and analysis

We used the Preferred Reporting Items for Systematic Reviews and Meta-analyses (PRISMA) checklist for reporting systematic reviews [25]. Because of clinical heterogeneity in the methodology, interventions, and outcome of the included studies, pooling of quantitative data to perform a meta-analysis was not possible and therefore a narrative synthesis was undertaken. To guide our conduct of narrative synthesis we consulted the “Guidance on the Conduct of Narrative Synthesis in Systematic Reviews” of Popay et al. [26].

To accomplish our secondary aim, the studies included in this systematic review were analyzed to detect patient characteristics associated with the use of a specific communication method. The algorithm was developed based on the associations found between characteristics and communication methods, and an algorithm published by Williams in 1992 [27]. During the construct, the algorithm was discussed in a local working group on communication with critically ill patients, consisting of intensivists, critical nurses, and a PhD student.

## Results

**Fig. 1:**
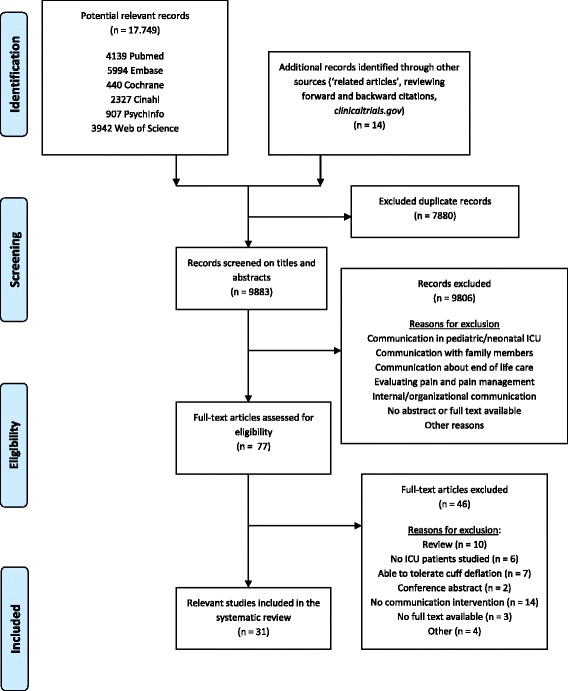
Flow diagram of the study selection procedure

Of the 9883 potentially relevant publications, 31 articles, representing 29 different studies, met all of the inclusion criteria. Two studies reported the results of their study in two different articles (the first article was about the study aim and design, the second article about the results). A flowchart illustrating the process of study selection is shown in Fig. 1. Characteristics of the included studies are summarized in Table 1. There were four studies with a quasi-experimental design [12, 28–30], 16 case series [31–46], four case reports [47–50], four pilot observational studies [19, 51–53], and a retrospective study [17]. We identified four communication intervention types: communication boards (three studies) [12, 17, 30]; speaking valves (eight studies) [36–40, 42, 48, 49]; electrolarynx (EL) (seven studies) [31, 33, 43, 44, 46, 47, 50]; and “high-tech” AAC (10 articles, representing nine studies) [19, 32, 34, 35, 41, 45, 51–54]. Three articles, representing two studies, studied multiple AAC interventions [28, 29, 55]. The outcomes reported contained a wide range of measures. Most commonly subjective assessments of improvement of communication or investigator-developed questionnaires were used.

**Table 1:** Characteristics of the included studies (*n* = 31)

Author, year	Study design and sample size	Intervention type	Study population (tracheostomized; intubated)	Measures	Main findings
Otuzoğlu, 2014 [12]	Quasi-experimental – no randomization – control group (*n* = 90)	Communication board	Orally intubated patients after cardiac surgery	Question forms for assessment: communication experiences during intubation period, and evaluation of communication process	Communication board was helpful (77.8 %; *n* = 35) and partially helpful (22.2 %; *n* = 10) In the control group 35.6 % (*n* = 16) had difficulties with the communication, in the treatment group 2.2 % (*n* = 1)
Patak, 2006 [17]	Retrospective study (*n* = 29) – no control group	Communication board	Patients with mixed diagnosis. Type of intubation unknown	Structured interview with 13 questions, self-developed: level of frustration without and if a communication board had been available	Frustration in communication would have been lower if a communication board had been offered (29.8 % vs 75.8 %; *p* < 0.001) 97 % (*n* = 28) perceived that a communication board would have been helpful in communicating effectively
Stovsky, 1988 [30]	Quasi-experimental – no randomization – control group (*n* = 40)	Communication board	Orally intubated patients after cardiac surgery	Open-end patient interview Satisfaction questionnaire Visual analog scale on satisfaction with communication	A planned communication with the board increased patient satisfaction (*t* = 2.09, *n* = 35, *p* = 0.05) 68 % (*n* = 27) in both groups stated that learning a communication technique before surgery was beneficial
Kluin, 1984 [36]	Case series (*n* = 19)	Speaking tube Portex “Talk”	Tracheostomized patients with mixed diagnosis	Subjective assessment of improved communication	74 % (*n* = 14) acquired intelligible speech, 16 % (*n* = 3) had fluctuating function due to problems with secretions or mental status, 11 % (*n* = 2) were unsuccessful
Kunduk, 2010 [37]	Case series (*n* = 10)	Speaking tube Blom Speech Cannula	Tracheostomized patients with mixed diagnosis	Success in phonation (e.g., sentence length and volume) Subjective satisfaction	90 % (*n* = 9) achieved sustained audible phonation and were very satisfied with the device and their speech quality
Leder, 2013 [40]	Case series (*n* = 23)	Speaking tube Blom Speech Cannula	Tracheostomized patients with mixed diagnosis	Voice intensity levels, obtained using a digital sound level meter Assessment of Intelligibility of Dysarthric Speakers (AIDS)	All participants achieved audible voicing Speech intelligibility scores improved from 80 % to 85 % (*p* = 0.03) Time to audible voicing was 6.60 min (SD 5.81)
Leder, 1990 [39]	Case series (*n* = 20)	Speaking tube Portex “Talk”	Tracheostomized patients with mixed diagnosis	Voice intensity levels, obtained using a digital sound level meter Subjective assessment by SLP	Significant greater voice intensity over ambient room noise at 5 l/min, 10 l/min, and 15 l/min (all *p* < 0.001) All subjects demonstrated adequate voice intensity for conversational speech intelligibility
Leder, 1989 [38]	Case series (*n* = 20)	Speaking tube Communi-Trach I	Tracheostomized patients with mixed diagnosis	See Leder, 1990 [39]	Significant greater voice intensity over ambient room noise at 5 l/min, 10 l/min, and 15 l/min (all *p* < 0.01) 90 % (*n* = 18) demonstrated adequate speech
Mitate, 2015 [48]	Case report (*n* = 1)	Speaking valve (Vocalaid)	Ventilator-dependent tetraplegic	Subjective assessment of improved communication	Talked 10 min with Vocalaid, with fatigue (inadequate for communication). Mouthstick stylus fixed on maxilla made communication possible with a communication board and an iPad touchscreen
Pandian, 2014 [49]	4 case reports (*n* = 4)	Speaking tube BLUSA cuffed tracheostomy tube	Tracheostomized patients with mixed diagnosis	Subjective assessment of improved communication	All achieved adequate phonation. One used it as his primary means of communication, the others only for short sentences to express their basic needs. With two cases someone else had to occlude the thumb port needed for phonation
Sparker, 1987 [42]	Case series (*n* = 19)	Speaking tube Portex “Talk” and Communi-Trach I	Tracheostomized patients, mainly with spinal cord fracture	Assessment of intelligibility with the AIDS (*n* = 5) Subjective assessment SLP (*n* = 19)	All patients were able to speak, 79 % (*n* = 15) utilized the device effectively for communication
Adler, 1986 [31]	Case series (*n* = 22)	Electrolarynx neck type	Tracheostomized patients with mixed diagnosis	Subjective assessment of improved communication by SLP (good, fair, or poor)	64 % (*n* = 14) achieved good results 14 % (*n* = 3) achieved fair results, 23 % (*n* = 5) achieved poor results
Ewing, 1975 [33]	Case series (*n* = 8)	Electrolarynx 1 neck type; 1 intra-oral type	Tracheostomized patients with unknown diagnosis	Daily written evaluation Patient and staff questionnaire to determine quality, ease of use, and preference for device	EL was preferred by both patients and staff over other communication methods (lip movement, sign language, writing) EL was: most comfortable, easiest to use, and clearest for self-expression
Girbes, 2014 [47]	Case report (*n* = 1)	Electrolarynx neck type	Orally intubated man after lung surgery	Subjective assessment of improved communication	EL enabled the patient to immediately produce intelligible speech
Shimizu, 2013 [50]	Case report (*n* = 1)	Electrolarynx neck type	Tetraplegic tracheostomized patient	Subjective assessment of improved communication	The patient gradually became better able to speak fluently and could be understood on the first day of EL use
Summers, 1973 [43]	Case series (*n* = 5)	Electrolarynx neck type	Tracheostomized patients with mixed diagnosis	Subjective assessment of improved communication	80 % (*n* = 4) was able to produce clear, intelligible speech 20 % (*n* = 1) was able to speak, but quite poorly Easy to learn how to use the device 60 % (*n* = 3) manipulated EL on the neck themselves
Tuinman, 2015 [44]	Case series (*n* = 15)	Electrolarynx	Mixed diagnosis. Oral tube (*n* = 13) and tracheostomy (*n* = 2)	A developed five-point Electrolarynx Effectivity Score (EES)	EL was effective or very effective (EES 4 and 5) with 40 % (*n* = 6). For two patients it improved lip-reading (EES 3) The intra-oral type was used successfully in one patient
Wu, 1974 [46]	Case series (*n* = 27)	Electrolarynx neck type	Diagnosis unknown. Oral (*n* = 4) and nasal tube (*n* = 4), tracheostomy (*n* = 19)	Subjective evaluation of improved communication (excellent, good, or failure)	70 % (*n* = 19) reported “excellent” or “good” results All excellent results came from tracheostomized patients Both nasotracheal and orotracheal intubated patients (*n* = 8) reported 50 % “good” results
Happ, 2004 [34]	Case series (*n* = 11)	“High-tech” AAC 2 VOCAs: – MessageMate – DynaMyte	Mixed diagnosis Oral tube (*n* = 7) and tracheostomy (*n* = 4)	Communication Methods Checklist Revised Ease of Communication Scale (ECS) Semi-structured interviews	ECS measurements showed significantly less difficulty with communication after device use (*t* > 2.62; *n* = 11, *p* = 0.047) 73 % (*n* = 8) used VOCAs with minimal assistance and instruction
Happ, 2005 [35]	Case series (*n* = 10)	“High-tech” AAC 2 VOCAs: – MessageMate – DynaMyte	Tracheostomized patients following surgical procedures for head or neck cancer	– Revised ECS – Semi-structured interviews – Questionnaires	VOCAs were used in 17 % (*n* = 8) of total observed events. 60 % of the messages were completed without assistance ECS scores showed less difficulty communicating with the VOCA (mean 19.8) in comparison with control group (mean 22.5)
Etchels, 2003 [32] MacAulay, 2002 [54]	Case series (*n* = 19)	“High-tech” AAC ICU-Talk communication computer	Mixed diagnosis. Oral tube (*n* = 13) and tracheostomy (*n* = 6)	ICU-Talk Project: Nurse Questionnaire Relative Questionnaire Patient Questionnaire	16 % (*n* = 3) remembered using it and found it useful Summary findings nurse questionnaires: 12 % used it as a first means of communication 44 % said ICU-Talk assisted with patient care 72 % said that the patient did stop using ICU-Talk
Garry, 2016 [51]	Pilot prospective study (*n* = 12) – no control group	“High-tech” AAC eye-tracking device The Tobii C12 eye-tracking computer	Mixed diagnosis. Oral tube (*n* = 3), tracheostomy (*n* = 8), self-maintained (*n* = 1)	Psychosocial Impact of Assistive Devices Scale (PIADS)	All patients were able to communicate basic needs to nursing staff and family. Positive mean overall impact score (PIADS = 1.30; *n* = 12, *p* = 0.004), and in mean scores for each PIADS domain: competence = 1.26, adaptability = 1.60, and self-esteem = 1.02 (all *n* = 12, *p* < 0.01)
Koszalinski, 2015 [52]	Pilot observational study (*n* = 20) – no control group	“High-tech” AAC Speak for Myself Computer Pad Software Application	Mixed diagnosis. Type of intubation unknown	Three open-ended questions that asked if the patient users liked or disliked using Speak for Myself	95 % (*n* = 19) stated that Speak for Myself was helpful for communication All but one patient said they would use Speak for Myself if hospitalized again and unable to speak Frustration was less with Speak for Myself (better able to communicate, more in control, more power to make choices)
Maringelli, 2013 [41]	Case series (*n* = 15)	“High-tech” AAC gaze-controlled communication system	Mixed diagnosis. Oral tube (*n* = 7) and tracheostomy (*n* = 8)	Internally developed pre- and post-intervention questionnaires, one per each group (patients, physicians, nurses)	Significant improvement in different communication domains, and a remarkable decrease of anxiety and dysphoric thought Improved the physicians’ and nurses’ ability to understand patients’ fundamental needs and clinical conditions (*p* < 0.001)
Miglietta, 2004 [53]	Pilot prospective study (*n* = 35) – no control group	“High-tech” AAC LifeVoice communication computer	Nonverbal acutely ill trauma patients Type of intubation unknown	Questionnaires with graded responses (1–5) related to ease of use and perception of improvement in comfort and anxiety (days 1, 3, and 7)	94 % (*n* = 33) of patients were interested in continued use >90 % of patients felt the system assisted them in obtaining their needs (pain management, hygiene, comfort, and anxiety) Hospital staff (*n* = 42) felt the device improved patient care (96 %) and comfort (91 %)
Rodriguez, 2012 [19]	Pilot observational study (*n* = 11) – no control group	“High-tech” AAC multifunctional communication computer	Patients mainly following surgery for head or neck cancer Type of intubation unknown	Usability of communication intervention form (every day) Patient satisfaction and usability instrument (prior to discharge)	Ability to independently use the device from day 1 until completion of the study 91 % (*n* = 10) of the participants were satisfied with use of the device and considered its use and functions of importance
Van den Boogaard, 2004 [45]	Case series (*n* = 9)	“High-tech” AAC “intelligent” keyboard compared with a letter board	Unknown diagnosis. Type of intubation unknown	Patient evaluation of satisfaction, convenience of use, and amount of effort required to work with each communication aid. Nurses were required to evaluate similarly	Both patients (88 % resp. 43 %) and nurses (86 % resp. 33 %) were more satisfied with the keyboard than the alphabetical letter board Five patients (56 %) thought the keyboard was easy to operate All patients chose to continue using it
Dowden, 1986a [28] Dowden, 1986b [55]	Quasi-experimental – no randomization – no control group (*n* = 50)	Divers AAC: – Oral (EL, speaking valve) – Fine motor (communication board, memowriter) – Limited switch (eye-scanning or single switch)	Mixed diagnosis. Type of intubation unknown	Interview before intervention for needs assessment (list of specific communication requirements) Interview after intervention to assess success of chosen communication aid	96 interventions were implemented with 50 patients. Motor control capabilities of patients allowed 49 % oral approaches, 46 % fine motor approaches, and 5 % limited switch approaches Oral approaches and fine motor approaches met an average of respectively 53 % and 68 % of their communication needs Use of several approaches simultaneously was most successful, this resulted in an average of 70–82 % of needs met
Happ, 2014 [29]	Quasi-experimental – no randomization – control group (*n* = 89)	Diverse AAC: Phase 1: usual care Phase 2: BCST Phase 3: training electronic AAC and consult SLP	Mixed diagnosis. Oral tube (*n* = 21) and tracheostomy (*n* = 68)	Frequency and quality of communication exchange Success of each communication exchange on a five-point scale Ease of communication by patient’s self-report on a five-point scale Frequency of AAC use per exchange	AAC was used in 0.84 % (Phase 1), 0.51 % (Phase 2), and 6.31 % (Phase 3) Increase in communication frequency in ICU Unit A (Phase 1 vs 3 (*p* < 0.0001); Phase 1 vs 2 (*p* < 0.0001)) Patients in the AAC + SLP intervention group used significantly more AAC methods (*p* = 0.002) and patients’ perceptions about communication ease improved (*p* < 0.01)

AIDS (Assessment of Intelligibility of Dysarthric Speakers): tool for quantifying single-word intelligibility, sentence intelligibility, and speaking rate of adult speakers with dysarthria

Revised ECS (Ease of Communication Scale): 10 Likert-type statements about perceived communication difficulty to patients who referred to a card printed in large font with response selections (0) not hard at all, (1) a little hard, (2) somewhat hard, (3) quite hard, (4) extremely hard

EES (Electrolarynx Effectivity Score): five-point scale: (1) no improved intelligibility, because of insufficient mouth movement; (2) no effect, but sufficient mouth movement;

(3) improved lip-reading by producing recognizable sounds; (4) effective, can speak words; (5) very effective, can make sentences

PIADS (Psychosocial Impact of Assistive Devices Scale): list of 26 self-reported items to assess functional independence, well-being, and quality of life

*AAC* augmentative and alternative communication, *VOCA* voice output communication aid, *SLP* speech language pathologist, *EL* electrolarynx, *BCST* basic communication skills training (e.g., communication board, writing)

### Quality assessment

Using the appraisal outlined, the overall methodological quality varied from poor to moderate; score 9–35 out of 42, median 17 (see Additional file 2). Overall the studies were small, with only two reporting an a-priori sample size calculation [12, 29]. Only six studies used measurement tools with statistical assessment of reliability and validity, the majority used a subjective assessment. Only four studies used comparator interventions [12, 28–30]. All studies had significant limitations in their design. There was a moderate to high risk of bias in the studies included in this review.

### Communication boards

A communication board for intubated patients consists of icons and pictures representing basic needs. This was used with three studies, one retrospective cohort and two quasi-experimental studies [12, 17, 30]. The first study, by Stovsky et al. (1988) [30], stated that a planned communication with a picture board (comprised of 22 pictures with words) increased patient satisfaction, measured with the visual analog scale on satisfaction with communication, in the early postoperative intubation period after cardiac surgery (*p* = 0.05). Of the patients, 70 % (*n* = 14) asked for items not indicated on the board [30]. The other two studies used a two-sided board with the alphabet, a picture of the human body, and a pain scale combined with sentences [17] or illustrations [12]. In the retrospective study by Patak et al. (2006) [17], the majority (97 %; *n* = 28) of patients reported in the structured interviews that the communication board would have been helpful in communicating effectively during mechanical ventilation and it would have decreased their frustration level (29.8 % vs 75.8 %, *p* < 0.001). The study by Otuzoğlu and Karahan(2014) [12] stated that for 77.8 % (*n* = 35) the illustrated communication material was beneficial for communication between the medical staff and the intubated patients. Of the patients in their intervention group, 91.1 % (*n* = 41) used the alphabetical part of the board. Advantages mentioned by all three studies were an increased efficiency and speed of communicating, decreased frustration, and quicker expression of patients their needs [12, 17, 30].

### Speaking tracheostomy tube with inflated cuff

Currently there are six types of specialized talking tracheostomy tubes available to allow communication with an inflated cuff [49]. The Portex Trach-Talk [36, 39, 42] and Communi-Trach I [42, 56] were used with four case series. The Portex BLUSA Tracheostomy Tube was reported in one article with four case reports [49]. These tracheostomy tubes have an additional lumen above the cuff through which air can flow into the larynx to facilitate verbal communication. Intelligible speech, measured by a subjective assessment of improvement of speech, was created in 100 % (*n* = 20) [39] and 74 % (*n* = 14) [36] using the Portex Trach Talk, in 90 % (*n* = 18) with the Communi-Trach I [56], and in 79 % (*n* = 15) using the Portex Trach-Talk and the Communi-Trach I (both or one of them were used) [42]. With the BLUSA tube, all patients (*n* = 4) achieved adequate phonation [49]. It took an average of 2.1 days (Portex “Talk”) [39] and 5.6 days (Communi-Trach I) [56] before adequate voice intensity for intelligible speech was produced.

The second option, reported in two case series, is the Blom Tracheostomy Tube which incorporates two separate valve mechanisms, through which all of the ventilator-delivered inspiratory air is directed to the lungs and the expiratory air can escape via fenestrations to the upper airway to allow phonation [37, 40]. With the Blom Tracheostomy tube, 90 % (*n* = 9) [37], measured with a subjective assessment, and 100 % (*n* = 23) [40], assessed with the Assessment of Intelligibility of Dysarthric Speakers, were able to achieve intelligible speech. In the study by Leder et al. (2013) [40], the time to audible voicing was 6.60 min. In both studies some of the subjects were also able to converse over the telephone [37, 40]. In the study by Kunduk et al. (2010) [37], two subjects (20 %) experienced clinically important oxygen saturation decreases (<90 %). The other study with the Blom Tracheostomy Tube showed no significant differences (*p* > 0.05) in oxygen saturation [40].

### Electrolarynx

The EL is a battery-powered handheld device which is pressed onto the skin of the neck to transmit the vibrated electronic sound into the oropharyngeal cavity, where the user modulates it to create speech via articulation [57]. We retrieved seven studies on usage of the EL in the ICU setting, of which five were case series [31, 33, 43, 44, 46] and two were case reports [47, 50]: 72 % (*n* = 57) of all subjects had a tracheostomy. Results showed successful communication, by creating intelligible speech, with the EL in patients with a tracheostomy in all of the case series: 86 % (*n* = 19) [31], 100 % (*n* = 8) [33], 80 % (*n* = 5) [43], 100 % (*n* = 2) [44], and 79 % (*n* = 15) [46]. Creating intelligible speech with the EL in intubated patients was observed for 50 % (*n* = 2) [46] and 46 % (*n* = 6) [44]. One study reported the use of an EL with a nasotracheal tube, of which 50 % (*n* = 2) had good results, meaning good value of the EL as a means of communication [46]. All but one of the studies measured the success of the EL with a subjective assessment of improved communication. Tuinman et al. (2015) used the self-developed Electrolarynx Effectivity Score. Both case reports reported successful use of the EL in creating intelligible speech with an intubated [47] and tracheostomized [50] patient. In the study by Ewing (1975) [33], the EL was the most preferred by the patients (*n* = 8) and staff (*n* = 32) over other available basic communication methods (lip movement, sign language, and writing). In the case series of Summers (1973) [43], clear intelligible speech was produced after 15–30 min of instruction in 60 % (*n* = 3) and 1–2 hours in 40 % (*n* = 2) of patients.

### High-tech communication intervention

All electronic AAC devices described in the nine studies had common topics about basic communication needs in the ICU on the main screens (e.g., emergency, pain, and emotions) [19, 32, 34, 35, 41, 45, 51–54]. Two case series reported the use of voice output communication aids (VOCAs), which are a subset of handheld AAC devices with which patients touch a word–picture icon on a keypad to produce a prerecorded voice message [34, 35]. VOCAs were used in 17 % [35] and 27 % [34] of observed communication events, and in both studies all patients were able to successfully generate valid messages. In 70 % [34] and 94 % [35] of the observed communication events, more than one method of communication was applied (e.g., gesture, mouthing words, head nods, and writing).

The computerized AAC devices are specialized computers that contain a database of prestored phrases or pictorials. The selected phrases are voiced by a speech synthesizer [19, 32, 41, 51–53]. The LiveVoice computer of the pilot study by Miglietta (2004) [53] uses various control devices for navigation through the menus; infrared eye-blink detector, touch buttons, or a touch-sensitive screen. Over 90 % of the patients felt that the system assisted them in obtaining their needs (pain management, hygiene, comfort, and anxiety). Of the hospital staff, 96 % (*n* = 42) felt that the LiveVoice improved patient care; this was not further specified. The pilot study by Rodriguez (2012) [19] used a multifunctional computer with touch buttons and a touch-sensitive screen. Ten patients (91 %) were satisfied with use of the device, measured with the Patient Satisfaction and Usability Instrument, and showed the ability to independently use the device from day 1. The use of the ICU-Talk communication computer was reported as a case series by Etchels et al. (2003) and MacAulay et al. (2002) [32, 54]. Of the patients who remembered using the computer, 16 % (*n* = 3) found it useful for creating conversations. Of the nurses, 44 % said the ICU-Talk assisted with patient care. The pilot study by Koszalinski et al. (2015) [52] with the Speak for Myself Computer Pad stated that 95 % (*n* = 19) thought the device was helpful for communication and it decreased frustration levels. The ‘intelligent’ keyboard of van den Boogaard and van Grunsven (2004) [45] scored more highly on ease of use (63 %) and satisfaction (88 %) compared with the alphabetical letter board. The gaze-controlled system of Maringelli et al. (2013) [41] improved significantly (*p* < 0.001) the ability to communicate basic needs and necessities, and decreased the level of anxiety remarkable. The eye-tracking device of Garry et al. (2016) [51] gave all of the patients the ability to communicate basic needs and had a positive psychosocial impact.

### Multiple AAC interventions

Dowden et al. (1986) [28, 55] described the use of AAC strategies for 50 temporarily nonspeaking ICU patients. The oral approaches (e.g., EL, speaking valve) and fine motor approaches (e.g., communication board) were the most recommended techniques. The most successfully served patients were those who were able to use several approaches simultaneously (70–82 % communication needs met). Reasons for intervention failure were decreased cognitive status (51 %), patient’s rejection of the intervention type (27 %), and decreased motor control (20 %).

The SPEACS trial by Happ et al. (2014) [29] measured the impact of two levels of interventions on communication interactions between nurses and intubated ICU patients (*n* = 89). They conducted a three-phase clinical trial: (1) usual care; (2) basic communication skills training; and (3) additional training in electronic AAC devices. Use of an AAC was 0.84 % (Phase 1), 0.51 % (Phase 2), and 6.31 % (Phase 3). The results demonstrated an increase in communication frequency in one ICU setting for both intervention groups (Phase 1 vs 3, *p* < 0.0001; and Phase 1 vs 2, *p* < 0.0001). The Phase 3 intervention added significant improvements to patients’ perceptions about communication ease (*p* < 0.01). No device limitations were mentioned.

### Patient characteristics and barriers for usage of the communication intervention

Patients using the communication boards were orientated without changes in mental status, able to see well enough to read the prints, and had no linguistic problems [12, 17, 30]. Two out of three studies used orally intubated patients after cardiac surgery [12, 30]. Difficulties with the use of the different communication boards were that the board contained too much information [17]. Certain needs and requirements of the patients were lacking on the board [12]. Also, optimal positioning of the board presented difficulties [30].

All tracheostomized patients in which the talking tracheostomy tubes were used were cognitively intact without upper-airway obstruction and had intact muscular function for articulation. Patients had mixed diagnoses [36–40, 42, 48, 49]. Common causes of malfunctioning of the tube included occlusion of the air vent ports, cuff leaks, and kinking of the airflow line tubing. One case report, also using a tracheostomy tube with additional lumen (Vocalaid), stated that it was inadequate for communication due to fatigue after a few minutes and discomfort [48].

The EL was used in patients with mixed diagnosis, intact cognition, and articulatory function [31, 33, 43, 44, 46, 47, 50]. Difficulties in using the EL were due to trouble of understanding the EL voice in the beginning and the unnatural voice quality. In four of the studies, some patients needed assistance in positioning of the device [31, 33, 44, 50].

High-tech communication interventions were used by patients who were cognitive able to communicate and follow simple commands [19, 32, 34, 35, 41, 45, 51–53]. In six studies patients needed to have some muscle power to use the device [19, 32, 34, 35, 45, 52]. Gaze-controlled and eye-tracking devices were used in paralyzed or physically limited patients with intact visual acuity [41, 51, 53]. Primary barriers to using the VOCAs were poor device positioning, deterioration of motor and/or cognitive function, and unfamiliarity of healthcare professionals with the use of the VOCA [34, 35]. Barriers to using the computerized AAC devices were fatigue, insufficient muscle power or coordination of the upper extremities, and reduced attention span or sedation [19, 32, 41, 45, 51–53].

### The algorithm

**Fig. 2:**
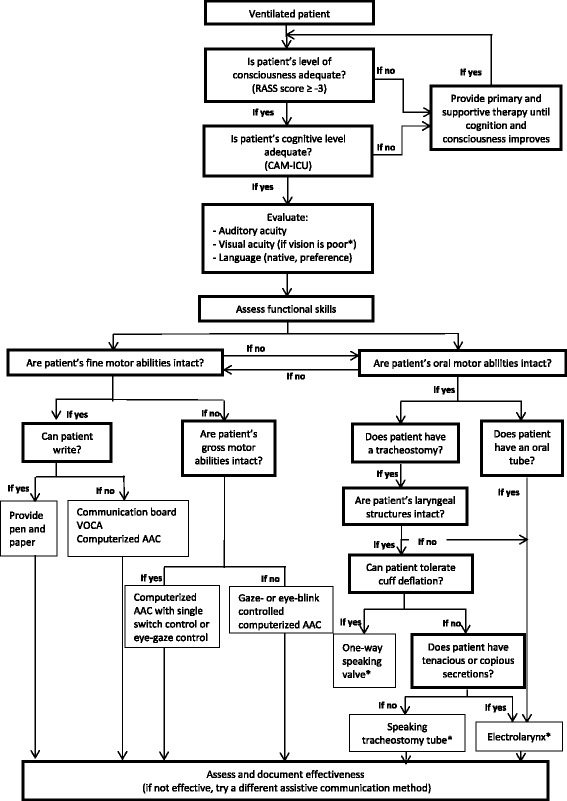
Algorithm for selecting alternative communication methods with intubated patients. *Able to use in patients with poor vision. *RASS* Richmond Agitation Sedation Scale, *CAM-ICU* Confusion Assessment Method for the ICU, *AAC* augmentative and alternative communication, *VOCA* voice output communication aid

To construct the algorithm we used the patient characteristics and the results which are presented in this systematic review. As a starting point we used the algorithm published by Williams in 1992 [27]. Through discussion, in the aforementioned working group, a hierarchy of assessment tasks to facilitate assessment and selection of communication methods was determined. The algorithm is shown in Fig. 2.

## Discussion

Our search of the literature revealed that relatively little attention in critical care research is given to the improvement of communication possibilities with ICU patients. The major finding of our systematic review on communication interventions for conscious and mechanically ventilated patients in the ICU is that in general all four communication intervention types—communication board, speaking valve, EL, and “high-tech” AAC devices—showed a demonstrable improvement in the patient’s ability to communicate; a strategy using a combination of communication methods is advisable. The enhanced communication through these devices may provide even small improvements in these vulnerable patients’ severe emotional reactions and contribution to healthcare decisions, thereby having a major impact on well-being.

The strengths of this review include the fact that is the first systematic review on the subject to our knowledge, the inclusion of all currently studied communication interventions for ventilated patients, the use of a robust search strategy to identify all studies on the matter, and the use of a validated quality assessment tool to evaluate the quality of the evidence. A limitation of the current state of evidence is that all of the included studies had poor to moderate methodological quality, which has important implications for the generalizability of the results. For example, there were no randomized controlled trials on the subject. A limitation of our results is that we could not conduct a meta-analysis, so no statements can be made on which communication method has proven to be most effective. Another threat to the validity of our results is publication and language bias. Also, when considering the results of the individual studies, the reader has to keep in mind that all patients were aware of participating in an experiment. It cannot be excluded that the increased patient satisfaction and positive outcomes with the communication tools reported could have been due to the increased attention they were receiving from the researchers and nurses, the so-called Hawthorne effect.

Recent data suggest that about 50 % of the patients in the ICU could be served by simple assistive communication tools [21]. The most straightforward method is the use of communication boards. One case report even presented the successful use of a mouthstick stylus (adjustable antenna) fixed on a mouthpiece with a communication board [48]. However, illustrated communication cannot fully comprehend the needs of patients. The specialized talking tracheostomy tubes can be a solution for tracheostomized patient who cannot tolerate cuff deflation. The Portex Trach-Talk was successfully used in four studies, [36, 38, 39, 42]; however, in another publication by Leder and Astrachan (1989) [56] stomal complications were reported in the form of pressure necrosis and wound extensions at the insertion of the airflow line. The more recently developed Portex BLUSA and the Blom Tracheostomy Tube appear to achieve sustained audible phonation [37, 40, 49]. Nonetheless, more studies are needed to assess safety and to decide whether they can be used as a primary means of communication. The EL seems to be another elegant device because it is easy to manipulate. Its effectiveness was mainly demonstrated with tracheostomized patients [31, 33, 43, 44, 46, 50], but it also seems to work with orally intubated patients [44, 46, 47]. An important advantage of most computerized AAC devices is that they can be equipped with different control devices to ensure that physical limitations do not prevent use of the device. None of the articles used the same type of electronic AAC, which makes it impossible to decide which software has the most potential.

Recent developments in mobile technology have provided interesting new tools for communication. Mobile communication apps are now available to enhance communication for individuals in the ICU [58–60]. These new devices need to be studied in future trials to define their effectiveness and role in communication with ventilated patients.

The main characteristics to limit usage of assistive communication materials, that were reported in the included studies, are the motor ability of the upper extremities (if needed to control the device), level of sedation, and cognitive status (cognitive fluctuation or deterioration) during critical illness. Also, reduced sensory status (e.g., lack of glasses or hearing aids) may be a barrier for effective communication.

A contributing factor in maintaining ineffective communication with intubated ICU patients is the absence of a systematic method of using various communication interventions [15, 18]. We believe that the use of communication interventions with intubated patients in the ICU needs to be imbedded in a communication strategy defined in a protocol. The strategy described in the protocol should be brief, minimally fatiguing, and immediately beneficial to both the patient and the staff [55, 61]. Secondly, ICUs need to be equipped with different low-end and high-end AAC devices to improve the ability to choose the most suitable intervention. Thirdly, healthcare professionals need to be trained in the usage of the various AAC devices. Lastly, it is important that the communication need and success of communication interventions are tracked in the medical chart of the patient. In this way, communication interventions will be tailored to the specific needs of speechless critically ill patients. Therefore, we developed an algorithm with a hierarchy of assessment tasks to facilitate the assessment and selection of a communication intervention with conscious mechanically intubated patients in the ICU. This algorithm is a starting point. Because the evidence for its design is scarce, it needs to be validated and possibly adjusted in clinical practice by health professionals and/or AAC experts. The algorithm could also be used for future studies.

The main question that still needs to be addressed is: what communication intervention works best for which ventilated patient? Further research with larger, multicenter studies is therefore needed to compare the effectiveness of the various communication techniques as well as introducing new innovative communications techniques. Future research should include specifics regarding baseline patient data, prerequired patient characteristics necessary to use the devices, level of sedation, training duration of staff and patients for usage of the communication device, and costs. Also, the use of different communication strategies needs to be studied.

## Conclusions

A summary of current available research on the various communication methods available for ventilated patients in the ICU is presented in this systematic review. The results of the four presented communication intervention types (communication board, speaking valve, EL, and “high-tech” AAC) all showed an improvement in the communication with mechanically ventilated patients. A combination of various methods may create the most effective communication option. However, the results should be interpreted with caution because evidence on the matter is limited and most studies did not have a comparator. Limitations in the use of assistive communication tools are the level of sedation, decreased cognitive function, and muscle power. We developed an algorithm to guide both clinical practice and further research for the use of assistive communication with intubated patients in the ICU.

## Additional files

Additional file 1: is a table presenting the detailed search strategy. (PDF 305 kb)
Additional file 2: is a table presenting critical appraisal assessment of the methodological quality of the studies using the Quality Assessment Tool (QATSDD); range 0–42). (PDF 287 kb)

## Abbreviations

AAC: Augmentative and alternative communication
AIDS: Assessment of Intelligibility of Dysarthric Speakers
BCST: Basic communication skills training
CAM-ICU: Confusion Assessment Method for the ICU
ECS: Ease of Communication Scale
EES: Electrolarynx Effectivity Score
EL: Electrolarynx
PIADS: Psychosocial Impact of Assistive Devices Scale
QATSDD: Quality Assessment Tool
RASS: Richmond Agitation Sedation Scale
SLP: Speech language pathologist
VOCA: Voice output communication aid
