# Supplementary material for: Communicating with conscious and mechanically ventilated critically ill patients: a systematic review
Source: Crit Care. 2016 Oct 19;20:333. doi: 10.1186/s13054-016-1483-2 (PMC5070186; doi:10.1186/s13054-016-1483-2)
Supplement: Additional file 1: — is a table presenting the detailed search strategy. (PDF 305 kb) [file 13054_2016_1483_MOESM1_ESM.pdf]

Additional file 1. Search Strategy

Database and Date of Search	Search Method		Results
**1. MEDLINE via Pubmed**<br><br>Date Search:<br>31-10-2015	#1	("Intensive Care"[Mesh:noexp]) OR ("Intensive Care Units"[Mesh:noexp]) OR ("Respiratory Care Units"[Mesh]) OR ("Respiration, Artificial"[Mesh]) OR ("Tracheostomy"[Mesh]) OR ("Intubation, Intratracheal"[Mesh]) OR ("Critical Care Nursing"[Mesh])	129695
	#2	(respiration[tiab] OR respirator therap*[tiab] OR respiratory therap*[tiab] OR respirator support[tiab] OR respiratory support[tiab] OR respirator assistance[tiab] OR respiratory assistance[tiab] OR ventilation[tiab] OR mechanical ventilat*[tiab] OR mechanically ventilat*[tiab] OR ventilator-dependent[tiab] OR ventilator dependent[tiab] OR tracheostom*[tiab] OR trachea stom*[tiab] OR intratracheal tube*[tiab] OR endotracheal tube*[tiab] OR intubat*[tiab] OR intensive care[tiab] OR ICU[tiab] OR ICUs[tiab] OR respiratory care[tiab] OR respiratory therapy[tiab] OR critical care[tiab] OR critically ill patient*[tiab]) OR (respiration[ot] OR respirator therap*[ot] OR respiratory therap*[ot] OR respirator support[ot] OR respiratory support[ot] OR respirator assistance[ot] OR respiratory assistance[ot] OR ventilation[ot] OR mechanical ventilat*[ot] OR mechanically ventilat*[ot] OR ventilator-dependent[ot] OR ventilator dependent[ot] OR tracheostom*[ot] OR trachea stom*[ot] OR intratracheal tube*[ot] or endotracheal tube*[ot] OR intubat*[ot] OR intensive care[ot] OR ICU[ot] OR ICUs[ot] OR respiratory care[ot] OR respiratory therapy[ot] OR critical care[ot] OR critically ill patient*[ot])	321242
	#3	"Nonverbal communication"[Mesh]	21651
	#4	(Communicat*[tiab] OR Nonverbal behavio*[tiab] OR Non-verbal behavio*[tiab] OR Non verbal behavio*[tiab] OR Non-vocal behavio*[tiab] OR Non vocal behavio*[tiab] OR Sign Language*[tiab] OR Body Language*[tiab] OR Gestur*[tiab] OR Facial expression*[tiab] OR Eye blink*[tiab] OR Lip read*[tiab] OR Head nod*[tiab] OR Alphabet board*[tiab] OR Word board*[tiab] OR Letter board*[tiab] OR Picture board*[tiab] OR Phrase board*[tiab] OR Electrolarynx[tiab] OR VOCA[tiab] OR VOCAs[tiab] OR augmentative device*[tiab] OR assisted vocalization[tiab] OR assisted speech[tiab]) OR (Communicat*[ot] OR Nonverbal behavio*[ot] OR Non-verbal behavio*[ot] OR Non verbal behavio*[ot] OR Non-vocal behavio*[ot] OR Non vocal behavio*[ot] OR Sign Language*[ot] OR Body Language*[ot] OR Gestur*[ot] OR Facial expression*[ot] OR Eye blink*[ot] OR Lip read*[ot] OR Head nod*[ot] OR Alphabet board*[ot] OR Word board*[ot] OR Letter board*[ot] OR Picture board*[ot] OR Phrase board*[ot] OR Electrolarynx[ot] OR augmentative device*[ot] OR assisted vocalization[ot] OR assisted speech[ot])	216911
	#5	(#1 OR #2) AND (#3 OR #4)	4688
	#6	Filters: English	**4139**
**2. Cochrane Library**<br><br>Date Search:<br>31-10-2015	#1	(Respiration or (respirator and therap*) or (respiratory and therap*) or "respirator support" or "respiratory support" or "respirator assistance" or "respiratory assistance" or ventilation or (mechanical and ventilat*) or (mechanically and ventilat*) or "ventilator-dependent" or "ventilator dependent" or tracheostom* or (trachea and stom*) or (intratracheal and tube*) or (endotracheal and tube*) or intubat* or "intensive care" or ICU or ICUs or "respiratory care" or "respiratory therapy" or "critical care" or ("critically ill" and patient*)):ab,ti,kw	47098
	#2	(Communicat* or (Nonverbal and behavio*) or ("Non-verbal" and behavio*) or ("Non verbal" and behavio*) or (Nonvocal and behavio*) or ("Non-vocal" and behavio*) or ("Non vocal" and behavio*) or (Sign and Language*) or (Body and Language*) or Gestur* or (Facial and expression*) or (Eye and blink*) or (Lip and read*) or (Head and nod*) or (Alphabet and board*) or (Word and board*) or (Letter and board*) or (Picture and board*) or (Phrase and board*) or Electrolarynx or VOCA or VOCAs or (augmentive and device*) or "assisted vocalization" or "assisted speech"):ab,ti,kw	11904
	#3	#1 AND #2	**440**

**3. EMBASE**<br><br><u>Date Search:</u><br>31-10-2015	#1	exp intensive care unit/ or exp artificial ventilation/ or exp assisted ventilation/ or exp tracheostomy/ or exp tracheostomy tube/ or exp trachea stoma/ or exp intensive care nursing/ or exp critically ill patient/	301319
	#2	((respiration OR (respirator therap*) OR (respiratory therap*) OR (respirator support) OR (respiratory support) OR (respirator assistance) OR (respiratory assistance) OR ventilation) OR (mechanical ventilat*) OR (mechanically ventilat*) OR (ventilator-dependent) OR (ventilator dependent) OR tracheostom* OR (trachea stom*) OR (intratracheal tube*) OR (endotracheal tube*) OR intubat* OR (intensive care) OR ICU OR ICUs OR (respiratory care) OR (respiratory therapy) OR (critically ill patient*) OR (critical care)).ti,ab	433485
	#3	exp nonverbal communication/ or exp facilitated communication/	33777
	#4	(Communicat* or (Nonverbal behavio*) or (Non-verbal behavio*) or (Non verbal behavio*) or (Nonvocal behavio*) or (Non-vocal behavio*) or (Non vocal behavio*) or (Sign Language*) or (Body Language*) or Gestur* or (Facial expression*) or (Eye blink*) or (Lip read*) or (Head nod*) or (Alphabet board*) or (Word board*) or (Letter board*) or (Picture board*) or (Phrase board*) or Electrolarynx or VOCA or VOCAs or (Augmentive device*) or (Assisted vocalization) or (assisted speech)).ti,ab.	269646
	#5	(#1 OR #2) AND (#3 OR #4)	8125
	#6	Limit (English language and embase)	**5994**
**4. PsychInfo**<br><br><u>Date Search:</u><br>31-10-2015	#1	(DE "Intensive Care") OR (DE "Artificial Respiration")	3343
	#2	(TI respiration or AB respiration) or (TI “respirator therap*” or AB “respirator therap*”) or (TI “respirator support” or AB “respirator support”) or (TI “respiratory support” or AB “respiratory support”) or (TI “respirator assistance” or AB “respirator assistance”) or (TI “respiratory assistance” or AB “respiratory assistance”) or (TI ventilation or AB ventilation) or (TI “mechanical ventilat*” or AB “mechanical ventilat*”) or (TI “mechanically ventilat*” or AB “mechanically ventilat*”) or (TI “ventilator-dependent” or AB “ventilator-dependent”) or (TI “ventilator dependent” or AB “ventilator dependent”) or (TI “tracheostom*” or AB “tracheostom*”) or (TI “trachea stom*” or AB “trachea stom*”) or (TI “intratracheal tube*” or AB “intratracheal tube*”) or (TI “endotracheal tube*” or AB “endotracheal tube*”) or (TI “intubat*” or AB “intubat*”) or (TI “intensive care” or AB “intensive care”) or (TI ICU or AB ICU) or (TI ICUs or AB ICUs) or (TI “respiratory care” or AB “respiratory care”) or (TI “respiratory therapy” or AB “respiratory therapy”) or (TI “critical care” or AB “critical care”) or (TI “critically ill patient*” or AB “critically ill patient*”)	13781
	#3	DE "Electronic Communication" OR DE "Computer Mediated Communication" OR DE "Nonverbal Communication" OR DE "Body Language" OR DE "Eye Contact" OR DE "Facial Expressions" OR DE "Gestures" OR DE "Manual Communication" OR DE "Verbal Communication" OR DE "Vocalization" OR DE "Speech Disorders" OR DE "Augmentative Communication" OR DE "Manual Communication" OR DE "Speech Therapy"	46844
	#4	(TI communicat* or AB communicat*) or (TI “Nonverbal behavio*” or AB “Nonverbal behavio*”) or (TI “Non-verbal behavio*” or AB “Non-verbal behavio*”) or (TI “Non verbal behavio*” or AB “Non verbal behavio*”) or (TI “Nonvocalbehavio*” or AB “Nonvocalbehavio*”) or (TI “Non-vocal behavio*” or AB “Non-vocal behavio*”) or (TI “Non vocal behavio*” or AB “Non vocal behavio*”) or (TI “Sign Language*” or AB “Sign Language*”) or (TI “Body Language*” or AB “Body Language*”) or (TI Gestur* or AB Gestur*) or (TI “Facial expression*” or AB “Facial expression*”) or (TI “Eye blink*” or AB “Eye blink*”) or (TI “Lip read*” or AB “Lip read*”) or (TI “Head nod*” or AB “Head nod*”) or (TI “Alphabet board*” or AB “Alphabet board*”) or (TI “Word board*” or AB “Word board*”) or (TI “Letter board*” or AB “Letter board*”) or (TI “Picture board*” or AB “Picture board*”) or (TI “Phrase board*” or AB “Phrase board*”) or (TI Electrolarynx or AB Electrolarynx) or (TI VOCA or AB VOCA) or (TI VOCAs or AB VOCAs) or (TI “augmentive device*” or AB “augmentive device*”) or (TI “assisted vocalization” or AB “assisted vocalization”) or (TI “assisted speech” or AB “assisted speech”)	173656
	#5	(#1 OR #2) AND (#3 OR #4)	945
	#6	Limiters - English	**907**

**5. CINAHL**<br><br>Date Search:<br>31-10-2015	#1	(MH "Respiratory Care Units") OR (MH "Critical Care Nursing+") OR (MH "Intensive Care Units") OR (MH "Post Anesthesia Care Units") OR (MH "Respiration, Artificial+") OR (MH "Intubation, Intratracheal+") OR (MH "Tracheostomy") OR (MH "Critically Ill Patients") OR (MH "Ventilator Patients")	59063
	#2	(TI respiration or AB respiration) or (TI “respirator therap*” or AB “respirator therap*”) or (TI “respirator support” or AB “respirator support”) or (TI “respiratory support” or AB “respiratory support”) or (TI “respirator assistance” or AB “respirator assistance”) or (TI “respiratory assistance” or AB “respiratory assistance”) or (TI ventilation or AB ventilation) or (TI “mechanical ventilat*” or AB “mechanical ventilat*”) or (TI “mechanically ventilat*” or AB “mechanically ventilat*”) or (TI “ventilator-dependent” or AB “ventilator-dependent”) or (TI “ventilator dependent” or AB “ventilator dependent”) or (TI “tracheostom*” or AB “tracheostom*”) or (TI “trachea stom*” or AB “trachea stom*”) or (TI “intratracheal tube*” or AB “intratracheal tube*”) or (TI “endotracheal tube*” or AB “endotracheal tube*”) or (TI “intubat*” or AB “intubat*”) or (TI “intensive care” or AB “intensive care”) or (TI ICU or AB ICU) or (TI ICUs or AB ICUs) or (TI “respiratory care” or AB respiratory care”) or (TI “respiratory therapy” or AB “respiratory therapy”) or (TI “critical care” or AB “critical care”) or (TI “critically ill patient*” or AB “critically ill patient*”)	48863
	#3	(MH "Assistive Technology Devices+") OR (MH "Nonverbal Communication+") OR (MH "Alternative and Augmentative Communication")	26965
	#4	(TI communicat* or AB communicat*) or (TI “Nonverbal behavio*” or AB “Nonverbal behavio*”) or (TI “Non-verbal behavio*” or AB “Non-verbal behavio*”) or (TI “Non verbal behavio*” or AB “Non verbal behavio*”) or (TI “Nonvocalbehavio*” or AB “Nonvocalbehavio*”) or (TI “Non-vocal behavio*” or AB “Non-vocal behavio*”) or (TI “Non vocal behavio*” or AB “Non vocal behavio*”) or (TI “Sign Language*” or AB “Sign Language*”) or (TI “Body Language*” or AB “Body Language*”) or (TI Gestur* or AB Gestur*) or (TI “Facial expression*” or AB “Facial expression*”) or (TI “Eye blink*” or AB “Eye blink*”) or (TI “Lip read*” or AB “Lip read*”) or (TI “Head nod*” or AB “Head nod*”) or (TI “Alphabet board*” or AB “Alphabet board*”) or (TI “Word board*” or AB “Word board*”) or (TI “Letter board*” or AB “Letter board*”) or (TI “Picture board*” or AB “Picture board*”) or (TI “Phrase board*” or AB “Phrase board*”) or (TI Electrolarynx or AB Electrolarynx) or (TI VOCA or AB VOCA) or (TI VOCAs or AB VOCAs) or (TI “augmentive device*” or AB “augmentive device*”) or (TI “assisted vocalization” or AB “assisted vocalization”) or (TI “assisted speech” or AB “assisted speech”)	56098
	#5	(#1 OR #2) AND (#3 OR #4)	2525
	#6	Limiters – English Language	**2327**
**6. Web of Science**<br><br>Date Search:<br>31-10-2015	#1	TS=(respiration OR (respirator NEAR/3 therap*) OR (respiratory NEAR/3 therap*) OR “respirator support” OR “respiratory support” OR “respirator assistance” OR “respiratory assistance” OR ventilation OR (mechanical NEAR/3 ventilat*) OR (mechanically NEAR/3 ventilat*) OR “ventilator-dependent” OR “ventilator dependent” OR tracheostom* OR (trachea NEAR/3 stom*) OR (intratracheal NEAR/3 tube*) OR (endotracheal NEAR/3 tube*) OR intubat* OR “intensive care” OR ICU OR ICUs OR “respiratory care” OR “respiratory therapy” OR “citical care” OR (“critically ill” NEAR/3 patient*))	332444
	#2	TS=(Communicat* or (Nonverbal NEAR/3 behavio*) or (“Non-verbal” NEAR/3 behavio*) or (“Non verbal” NEAR/3 behavio*) or (Nonvocal NEAR/3 behavio*) or (“Non-vocal” NEAR/3 behavio*) or (“Non vocal” NEAR/3 behavio*) or (Sign NEAR/3 Language*) or (Body NEAR/3 Language*) or Gestur* or (Facial NEAR/3 expression*) or (Eye NEAR/3 blink*) or (Lip NEAR/3 read*) or (Head NEAR/3 nod*) or (Alphabet NEAR/3 board*) or (Word NEAR/3 board*) or (Letter NEAR/3 board*) or (Picture NEAR/3 board*) or (Phrase NEAR/3 board*) or Electrolarynx or VOCA or VOCAs or “assisted vocalization” or “assisted speech”)	486716
	#3	#1 AND #2	4174
	#4	**LANGUAGE:** (English)	**3942**
