# Supplementary material for: Communicating with conscious and mechanically ventilated critically ill patients: a systematic review
Source: Crit Care. 2016 Oct 19;20:333. doi: 10.1186/s13054-016-1483-2 (PMC5070186; doi:10.1186/s13054-016-1483-2)
Supplement: Additional file 2: — is a table presenting critical appraisal assessment of the methodological quality of the studies using the Quality Assessment Tool (QATSDD); range 0–42). (PDF 287 kb) [file 13054_2016_1483_MOESM2_ESM.pdf]

Additional file 2. Critical Appraisal (QATSDD) (range 0-42)

Criteria	Commu-nication boards			Speaking valve								Electrolarynx							"High-tech" AAC									Divers		% of maximum possible score obtained by all papers
	Otuzoglu, 2013	Patak, 2006	Stovsky, 1988	Kluin, 1984	Kunduk, 2010	Leder, 1990	Leder, 1989	Leder, 2013	Mitate, 2015	Pandian, 2014	Sparker, 1987	Adler, 1986	Ewing, 1975	Girbes, 2014	Shimizu, 2013	Summers, 1973	Tuinman, 2014	Wu, 1974	Happ, 2004	Happ, 2005	Etchels, 2003	Garry, 2015	Koszalkinski, 2014	Maringelli, 2013	Miglietta, 2004	Rodriguez, 2012	Van den Boogaard, 2003	Dowden, 1986a,b	Happ, 2014	
**1. Explicit theoretical framework**	3	3	2	2	2	2	2	3	2	2	1	1	1	1	1	2	2	2	3	3	2	3	3	2	2	2	2	1	2	**68**
**2. Statement of aims/objectives in main body of report**	2	3	3	2	2	2	2	2	3	3	1	1	2	2	2	2	2	2	3	3	1	3	1	3	2	3	1	3	3	**74**
**3. Clear description of research setting**	3	2	3	1	3	1	1	2	1	1	2	2	1	1	3	1	2	3	3	3	2	3	1	3	3	3	1	2	3	**69**
**4. Evidence of sample size considered in terms of analysis**	2	0	0	0	0	0	0	0	0	0	0	0	0	0	0	0	0	0	0	0	0	0	0	0	0	0	0	0	3	**6**
**5. Representative sample of target group of reasonable size**	1	1	1	1	1	1	1	1	1	1	1	1	1	1	1	1	1	1	1	1	1	1	1	1	1	1	1	1	2	**34**
**6. Description of procedure for data collection**	2	2	3	1	2	3	3	3	1	1	2	2	1	1	2	2	1	2	2	2	2	2	1	2	2	3	3	2	3	**67**
**7. Rationale for choice of data collection tool(s)**	0	0	1	0	0	1	1	1	0	0	2	0	1	0	0	0	1	0	2	2	1	2	0	2	1	2	0	1	2	**26**
**8. Detailed recruitment data**	1	3	3	1	2	2	2	2	2	1	1	2	1	2	2	2	1	1	2	2	2	1	1	1	2	2	2	1	3	**57**
**9. Statistical assessment of reliability and validity of measurement tool(s) (Quantitative only)**	0	#	2	0	0	0	0	0	#	#	0	0	0	0	0	0	0	0	2	2	0	1	#	0	#	1	0	0	3	**15**
**10. Fit between stated research question and method of data collection (Quantitative only)**	2	#	2	1	1	2	2	2	#	#	2	2	1	0	0	1	2	1	2	2	2	2	#	2	#	2	1	2	2	**53**
**11. Fit between stated research question and format and content of data collection tool e.g. interview schedule (Qualitative only)**	X	2	X	X	X	X	X	X	1	1	X	X	X	X	X	X	X	X	2	2	X	X	1	X	2	X	X	X	0	**46**
**12. Fit between research question and method of analysis**	2	1	2	1	2	2	2	2	1	1	1	1	1	0	0	1	2	1	3	3	1	2	1	2	1	3	1	2	3	**52**
**13. Good justification for analytical method selected**	0	1	0	0	0	0	0	0	0	0	0	0	0	0	0	0	0	0	1	1	0	2	0	0	0	1	0	1	3	**11**
**14. Assessment of reliability of analytical process (Qualitative only)**	X	2	X	X	X	X	X	X	0	0	X	X	X	X	X	X	X	X	2	2	X	X	0	X	0	X	X	X	0	**25**
**15. Evidence of user involvement in design**	0	0	0	0	0	0	0	0	0	0	0	0	0	0	0	0	0	0	0	0	0	0	0	0	0	0	0	0	0	**0**
**16. Strengths and limitations critically discussed**	2	1	3	2	2	2	2	2	2	2	2	2	2	1	2	2	1	2	2	2	1	3	1	2	2	2	2	1	3	**63**
**Total score**	**20**	**21**	**25**	**12**	**17**	**18**	**18**	**20**	**14**	**13**	**15**	**14**	**12**	**9**	**13**	**14**	**15**	**15**	**30**	**30**	**15**	**25**	**11**	**20**	**18**	**25**	**14**	**17**	**35**	
**% of highest possible score**	**48**	**50**	**60**	**29**	**40**	**43**	**43**	**48**	**33**	**31**	**36**	**33**	**29**	**21**	**31**	**33**	**36**	**36**	**63**	**63**	**36**	**60**	**26**	**48**	**43**	**60**	**33**	**40**	**73**	

Criteria were scored on a scale from 0 to 3 (0 = not at all, 1 = very slightly, 2 = moderately, 3 = complete, # = no quantitative methods used, X = no qualitative methods used); AAC = Augmentative and Alternative Communication
